# Infection with SARS-CoV-2 is associated with menstrual irregularities among women of reproductive age

**DOI:** 10.1371/journal.pone.0276131

**Published:** 2022-10-26

**Authors:** Emily M. Cherenack, Ana S. Salazar, Nicholas F. Nogueira, Patricia Raccamarich, Violeta J. Rodriguez, Alejandro M. Mantero, Allison Marsh, Sophia Gerard, Marissa Maddalon, Deborah L. Jones, Nichole R. Klatt, Maria L. Alcaide

**Affiliations:** 1 Department of Public Health Sciences, University of Miami Miller School of Medicine, Miami, Florida, United States of America; 2 Department of Medicine, University of Miami Miller School of Medicine, Miami, Florida, United States of America; 3 Department of Psychology, University of Georgia, Athens, Georgia, United States of America; 4 Department of Psychiatry and Behavioral Sciences, University of Miami Miller School of Medicine, Miami, Florida, United States of America; 5 Department of Surgery, University of Minnesota Medical School, Minneapolis, Minnesota, United States of America; 6 Department Obstetrics and Gynecology, University of Miami Miller School of Medicine, Miami, Florida, United States of America; Washington State University, UNITED STATES

## Abstract

**Background:**

Biological and psychological mechanisms may be responsible for menstrual irregularities occurring among women during the COVID-19 pandemic.

**Study design:**

From January 2019 to September 2021, women (18- to 45-years-old and not using hormonal contraception) were recruited in Miami-Dade County, Florida. Cross-sectional, self-report surveys collected data on menstrual irregularities, COVID-19 vaccination, stress, depression, and loneliness. A EUA approved rapid test assay using whole blood measured SARS-CoV-2 IgG antibodies. Chi-square and Fisher’s exact tests described menstrual irregularities among women recruited before versus after the start of the COVID-19 pandemic and with detectable versus undetectable SARS-CoV-2 IgG antibodies. A logistic regression examined the relationship between the presence of SARS-CoV-2 IgG antibodies and menstrual irregularities controlling for age, stress, depression, and loneliness.

**Results:**

Among 182 women enrolled, 73 were enrolled after pandemic onset, and 36 provided vaccination data. Having detectable SARS-CoV-2 IgG antibodies was associated with a higher percentage of menstrual irregularities among unvaccinated women (0% vs. 39%, *p* = .026) and among all women regardless of vaccination status (31% vs. 5%; *p* = .005). Adjusting for age and psychological variables, the odds of menstrual irregularities were 7.03 times (95% CI [1.39, 35.60]; *p* = .019) higher among women with detectable antibodies compared to women without detectable antibodies. Neither enrollment date, age, nor psychological factors were associated to menstrual irregularities.

**Conclusions:**

Biological mechanisms related to SARS-CoV-2 infection may be responsible for irregular menstruation and should be further examined to mitigate the impact of the COVID-19 pandemic on women’s health.

## Introduction

In January 2020, the World Health Organization declared an international public health emergency in response to the novel coronavirus SARS-CoV-2 [1]. COVID-19, the disease caused by SARS-CoV-2, was named a global pandemic in March 2020 [1]. As of April 2022, it is estimated that over 80 million individuals have been infected with SARS-CoV-2, and nearly 983,000 people have died in the US as a result of COVID-19 [2]. In addition to COVID-19, SARS-CoV-2 infection and pandemic-related stressors have affected physical health, mental health, and economic well-being at a population level [3, 4]. Moreover, the pandemic has uniquely impacted women and those from historically marginalized racial and ethnic communities, thereby widening existing health disparities driven by discrimination and sociocultural factors [5–8].

Compared to men, women may be more likely to experience post-acute sequalae of SARS-CoV-2 infection affecting multi-organ systems and mental health [9]. Pregnant women with SARS-CoV-2 infection are at higher risk for adverse pregnancy and birth-related outcomes [10, 11]. Women also report greater specific pandemic-related stressors, such as loss of child-care, higher stress, and loneliness [12]. Despite women being disproportionally affected by the pandemic, there is lack of research evaluating the impacts of the COVID-19 pandemic on women’s health.

Menstruation is one factor unique to the health of women of reproductive age. Menstrual regularity can be impacted by hormonal birth control, hormonal and metabolic dysregulation (e.g., as occurs in polycystic ovary syndrome), and poor nutritional intake in the context of high energy expenditure, which may differ by social constructs such as race or income [13–17]. Recent studies suggest that menstruation may also be disrupted during the COVID-19 pandemic [18–22]. However, it is unknown to what extent disruptions are a result of stress, mental health, infection with SARS-CoV-2, and/or COVID-19 vaccination. Although some pre-pandemic research shows a link between stress and menstrual irregularities, there are conflicting findings about the strength and directionality of this relationship [23–26]. Research on mental health and menstruation during the COVID-19 pandemic has also shown mixed results. Among women of reproductive age in Turkey, COVID-19 related stress, depression, and anxiety have been associated with menstrual irregularities and somatic menstrual symptoms (e.g., bloating, fatigue) [18, 22]. Women in the US with high COVID-related stress are also more likely to report changes in menstrual duration and heavy menstrual bleeding compared to women with moderate stress [20]. However, other studies have failed to find changes to menstruation or an association between stress and menstruation during the COVID-19 pandemic [27].

Biological mechanisms associated with SARS-CoV-2 infection may also drive menstrual irregularities. In Shanghai, women hospitalized with COVID-19 reported disruptions to their menstrual cycles after infection, including a decrease in menstrual volume (20%) and prolonged menstrual cycles (20%), and it has been suggested that changes in the menstrual cycle may be related to the presence of specific proteases in the female reproductive tract and the ability for SARS-CoV-2 to infect endometrial cells [19, 28]. In addition, there have been anecdotical reports of menstrual irregularities after COVID-19 vaccination, and it is unknown whether menstrual irregularities are caused solely by vaccination or if they are clinically significant [29, 30]. In one study, after vaccination against COVID-19, women reported an increase in the length of the menstrual cycle of less than one day [31]. Another study showed increases in heavy menstrual bleeding after COVID-19 vaccination, which remitted in about two months [32].

The existing research on menstruation during the COVID-19 pandemic has not focused on historically marginalized racial or ethnic groups, which fails to address the disproportionate impacts of the COVID-19 pandemic among these communities in the US who are more likely to have pre-existing COVID-19 risk factors, to be infected with SARS-CoV-2, and to die from COVID-19 [5, 8, 21, 33]. Moreover, income loss during the COVID-19 pandemic has been associated with menstrual product insecurity [34]. COVID-19 may thereby perpetuate existing economic disparities and negatively impact women’s menstrual experiences and quality of life [35–37]. To fully understand reproductive health during the COVID-19 pandemic, it is critical to identify factors related to menstrual irregularities in a sample that includes women from historically marginalized racial and ethnic groups.

The aim of this study was to evaluate menstrual irregularities during the COVID-19 pandemic among a sample of mostly Black and Hispanic women and use a biopsychosocial perspective to simultaneously examine the relationships of SARS-CoV-2 infection and/or COVID-19 vaccination and psychological factors with menstrual irregularities.

## Methods

### Recruitment

**Fig 1:**
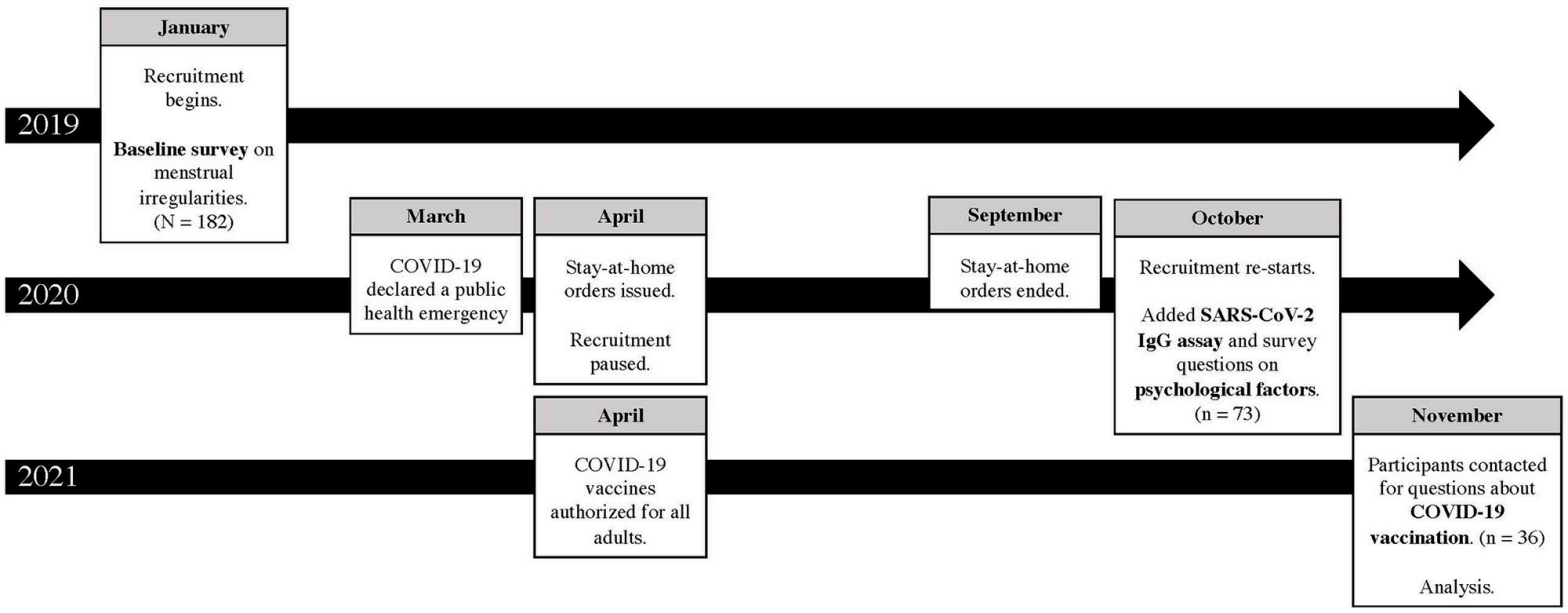
Study timeline. This research was conducted in Miami-Dade County in South Florida. In March 2020, COVID-19 was declared a public health emergency in Florida. From April 2020 to September 2020, stay-at-home orders were issued for the state. In April 2021, vaccines against COVID-19 were authorized for all residents over 18.

From January 2019 to September 2021, cisgender women of reproductive age were enrolled in a study evaluating behavioral and biological mechanisms underlying bacterial vaginosis and HIV risk; the current analysis includes cross-sectional data collected as part of a baseline assessment. Women were recruited via word of mouth, community flyers, the Center for AIDS Research, and the Center for HIV and Research in Mental Health in Miami-Dade, Florida. Inclusion criteria included being a cisgender woman between 18–45 years old, reporting sexual activity in the past three months, being confirmed HIV-negative, no intrauterine device, no history of surgical treatment of the cervix or cervical intra-epithelial neoplasia grade 2 or 3, no chlamydia or gonorrhea in the past two months, no antibiotic use in the past two months, and no allergy to metronidazole. In the current analysis, women were excluded for factors that could impact menstruation, including taking hormonal contraceptives, female hormones, or pregnancy within the last year. Fig 1 presents a timeline of study activities in the context of the COVID-19 pandemic. All women were recruited either prior to the pandemic or after stay-at-home orders for Florida had ended.

### Measures

Procedures were in accordance with the ethical standards of the responsible committee on human experimentation and the Helsinki Declaration of the World Medical Association. Ethical approval was received from the Institutional Review Board at the University of Miami (#20180758). All participants provided written informed consent. Participants completed a self-report survey that measured menstrual irregularities over the past three months (“have you had regular menstrual cycles (26–35 days) in the three months prior?”) and sociodemographic measures, including age, average monthly income, educational attainment, employment status, race (i.e., Asian, Black, Native American, Native Hawaiian or Pacific Islander, other, and/or white), and ethnicity (i.e., Haitian, Hispanic, non-Hispanic, and/or other).

Beginning October 2020, participants received a Food and Drug Administration (FDA) Emergency Use Authorization (EUA) rapid chromatographic immunoassay for the qualitative detection of SARS-CoV-2 IgG antibodies in whole blood (Cliawaived Inc. CoronaCHEK COVID-19 Rapid Test Kit). Detectable antibodies are suggestive of either previous SARS-CoV-2 infection and/or vaccination against COVID-19. Validated psychological measures added to the survey at this time included the 4-item Perceived Stress Scale (Cronbach’s α = .71), 10-item Centers for Epidemiological Studies Depression Scale (CES-D-10) (Cronbach’s α = .85), and the 3-item UCLA Loneliness Scale (Cronbach’s α = .81), which were summed to calculate continuous scores for perceived stress, depression symptom severity, and loneliness, respectively [38–40]. In November 2021, participants were asked if and when they had received a vaccination for COVID-19.

### Analysis

Descriptive analyses evaluated sample characteristics. The percent of the sample with detectable SARS-CoV-2 IgG antibodies, the percent reporting irregular menstruation, and mean scores for perceived stress, depression, and loneliness were examined across time when separated into month and year of recruitment; however, due to the small number of participants recruited each month, we were underpowered to test for the significant differences in factors by recruitment date.

Chi-square tests of significant differences were used to examine if the proportion of women reporting menstrual irregularities differed between women who completed the survey pre-pandemic (January 2019 to March 2020) compared to women who completed the survey during the pandemic (October 2020 to November 2021), which was after a pause in research activities from April to September 2020. Using Fisher’s Exact Test to account for small cell sizes, the association between detectable SARS-CoV-2 IgG antibodies and menstrual irregularities was examined in the sub-sample of participants who confirmed they had not received a COVID-19 vaccination (in which case having detectable IgG antibodies was indicative of prior SARS-CoV-2 infection). Chi-square tests also explored whether there was a relationship between having detectable SARS-CoV-2 IgG antibodies and menstrual irregularities among all participants (i.e., including unvaccinated women, vaccinated women, and women with unknown vaccination status). Three linear regressions were conducted to examine associations between SARS-CoV-2 IgG antibodies and perceived stress scores, depression scores, and loneliness scores.

Among all women recruited after the start of the pandemic, a multivariable logistic regression tested if the presence of SARS-CoV-2 IgG antibodies was associated with menstrual irregularities when controlling for age, perceived stress scores, depression scores, and loneliness scores. Linearity assumptions in logistic regression were assessed using box Tidwell test, interactions using log-likelihood ratio tests, and goodness-of-fit using Hosmer-Lemeshow test. Confounding was indicated by a 10% change in Odds Ratio (OR). Condition indexes (CNIs) and variance decomposition proportion (VDP) values were also examined to identify multicollinearity issues. Individuals with missing data on outcome and/or covariates were excluded from the regression analysis (i.e., two participants had missing data on the CES-D). Alpha was set to *p* = .05. Analyses were conducted using SPSS 28 & SAS 9.4.

## Results

### Sample description

A total of 182 participants completed the baseline assessments (i.e., demographics, menstrual irregularities). Of those, 73 completed measures implemented after the COVID-19 pandemic was established (i.e., SARS-CoV-2 IgG antibody assay, stress, depression, loneliness). Thirty-six participants responded to a follow-up questionnaire on prior COVID-19 vaccination.

Sociodemographic characteristics, psychological factors, presence of SARS-CoV-2 IgG antibodies, history of COVID-19 vaccination, and menstrual irregularities are illustrated in Table 1. Most of the sample consisted of women from historically minoritized racial and ethnic groups, with only 16 (9%) identifying as non-Hispanic white. Half (50%) of participants had not attended college, over half were unemployed (57%), and half (51%) made less than $1000 in monthly household income.

**Table 1:** Sociodemographics, psychological factors, SARS-CoV-2 IgG antibodies, history of COVID-19 vaccination, and menstrual irregularities.

**Age** (*n* = 182)	*M*^a^ = 33 (*SD*^b^ = 8)
**Race** (*n* = 182)	*n* (%)
Asian	2 (1)
Black	108 (59)
Native American	2 (1)
Other	10 (6)
White	60 (33)
**Ethnicity** (*n* = 182)	
Haitian	7 (4)
Hispanic	55 (30)
Non-Hispanic	94 (52)
Other	26 (14)
**Highest Level of Education**	
Grades 1–11	29 (16)
High school / GED	62 (34)
Some college / associates degree	48 (26)
Four-year college	29 (16)
Attended or completed graduate school	14 (8)
**Employment**	
Employed	78 (43)
Unemployed	104 (57)
**Monthly Income**	
$500 or less	54 (30)
$500–$1000	38 (21)
$1001–$3000	58 (32)
$3001–$6250	17 (9)
More than $6250	14 (8)
**Psychological Factors**	
Mean Perceived Stress Score (PSS-4^c^) (*n* = 73)	M = 9.6 (SD = 3.6)
Mean Depression Score (CES-D-10 ^d^) (*n* = 71)	M = 9.4 (SD = 6.9)
Mean Loneliness Score (UCLA-Loneliness Scale-3)	M = 5.2 (SD = 1.9)
**SARS-CoV-2 IgG Antibodies** (*n* = 73)	
Undetectable IgG	37 (51)
Detectable IgG	36 (49)
**COVID-19 Vaccination** (*n* = 36)	
Vaccinated (at least 1 dose)	7 (19)
Unvaccinated	29 (81)
With detectable antibodies	18 (62)^e^
**Menstrual irregularities** (*N* = 182)	25 (14)

^a^Mean,

^b^Standard Deviation,

^c^Perceived Stress Scale,

^d^Center for Epidemiological Studies Depression Scale,

^e^Percent of unvaccinated participants with detectable antibodies.

**Fig 2:**
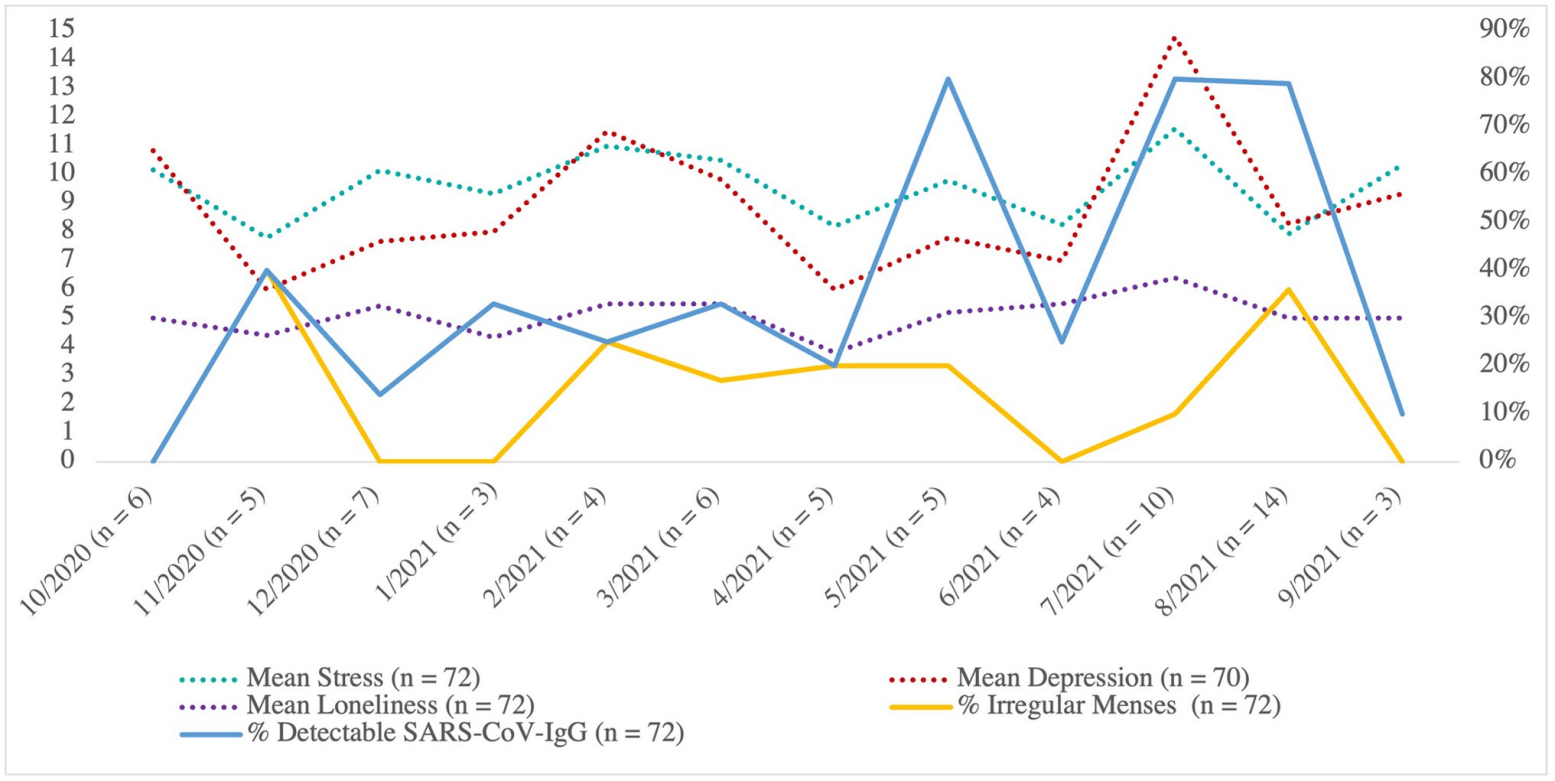
SARS-CoV-2 IgG antibodies, menstrual irregularities, and psychological factors over time. Data collected after the start of the COVID-19 pandemic on the percent of participants with detectable IgG antibodies, percent of participants with menstrual irregularities, and mean scores on perceived stress as measured by the Perceived Stress Scale, depression as measured by the Center for Epidemiological Studies Depression Scale, and loneliness as measured by the UCLA Loneliness Scale were calculated separately for each month of recruitment. Dotted lines are used for variables calculated as mean scores (continuous scale shown in left vertical axis). Solid lines are used for dichotomous variables presented as a percentage of the sample with the characteristic (percentage shown in right vertical axis).

For psychological variables, 31 (43%) participants endorsed at least one item indicative of perceived stress (e.g., felt difficulties were piling up so high that you could not overcome them) from “fairly often” to “very often” over the last month. Twenty-eight (38%) participants met the cut-off criteria suggestive of elevated depression, defined as 10 or more on the CES-D [38]. On the UCLA Loneliness Scale, 40 (55%) fell in the “not lonely” range, while 33 (45%) fell in the “lonely” range [40]. Fig 2 shows the percent of participants with detectable SARS-CoV-2 IgG antibodies, the percent of women reporting irregular menstruation, and mean stress, depression, and loneliness over time when separated by recruitment month and year.

Of the 36 participants who responded to a follow-up survey on COVID-19 vaccination, seven participants had received vaccination against COVID-19. This suggests most participants with detectable antibodies had SARS-CoV-2 prior infection.

### Bivariate analyses

Across the entire sample (N = 182), 25 (14%) of participants reported menstrual irregularities over the past three months. Among women who confirmed they had not been vaccinated, Fisher’s exact test showed a significant relationship between having detectable SARS-CoV-2 IgG antibodies and menstrual irregularities: all seven unvaccinated women reporting menstrual irregularities had detectable SARS-CoV-2 antibodies (39% irregular menstruation among women with detectable antibodies vs. 0% among undetectable, *p* = .026) (Table 2). In the larger sample including vaccinated, unvaccinated, and unknown-status participants, a Chi-square test of independence showed women who had detectable SARS-CoV-2 antibodies were significantly more likely to report menstrual irregularities (31% vs. 5%, *p* = .005). Group comparisons of women reporting menstrual irregularities prior to pandemic start and during the pandemic were not significant. In three separate linear regressions, there were no significant bivariate associations between SARS-CoV-2 IgG antibody status and perceived stress (*p* = .279), depression (*p* = .690), or loneliness (*p* = .427).

**Table 2:** Bivariate associations between recruitment date and SARS-2-CoV antibodies and menstrual irregularities.

Variables Being Compared	Menstruation		Chi-Square Tests of Significant Differences
	*n* (%)		
	Irregular	Regular	
**Recruitment date** (*N* = 182)			*X*^2^ (1, *N* = 182) = 1.706, *p* = .192
Pre-pandemic (*n* = 109)	12 (11)	97 (89)	
During pandemic (*n* = 73)	13 (18)	60 (82)	
**Antibody status** (*N* = 73)			***X***^**2**^ **(1, *N* = 73) = 7.885, *p* = .005**^a^
Detectable IgG (*n* = 36)	11 (31)	25 (69)	
Undetectable IgG (n = 37)	2 (5)	35 (95)	
**Antibody status, unvaccinated only** (*N* = 29)			Fisher’s Exact Test, ***p* = .026**
Detectable IgG (*n* = 18)	7 (39)	11 (61)	
Undetectable IgG (*n* = 11)	0 (0)	11 (100)	

^a^Tests with p-values below .05 are bolded.

### Multivariable logistic models

**Fig 3:**
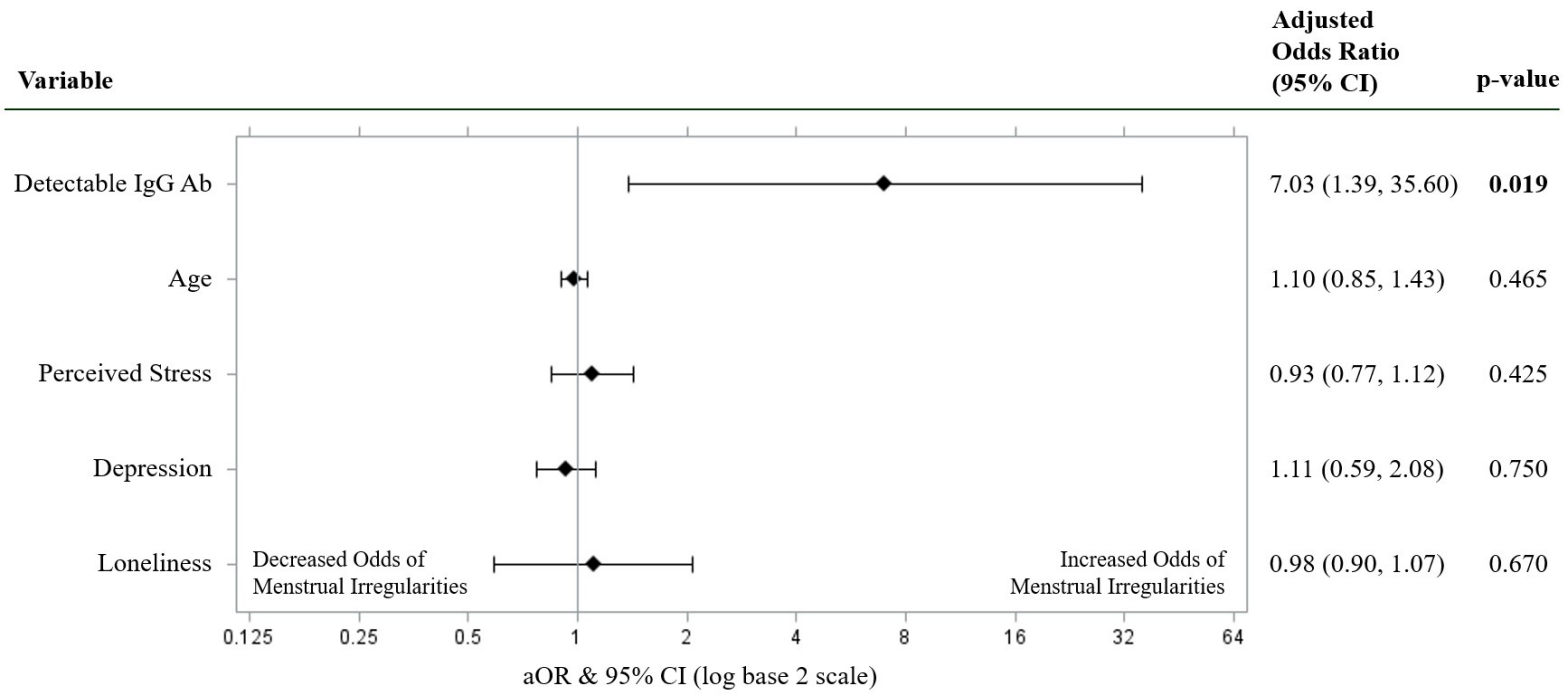
Adjusted odds ratio for menstrual irregularities. Predictors of menstrual irregularities in a multivariable logistic regression model containing detectable IgG antibodies, age, perceived stress score, depression score, and loneliness score. Adjusted Odd Ratios of menstrual irregularities were calculated and presented using a log base 2 scale in a Forest Plot. Null line is indicated for no predictor effects and bolded lines represent adjusted odds ratio with 95% confidence intervals. Bolded lines above and below the null line indicate increased or decreased odds of menstrual irregularities, respectively. *Note*. Detectable IgG Ab were operationalized into Detectable/Undetectable SARS-COV-2 IgG Antibodies.

Fig 3 presents results for multivariable models investigating the relationship of SARS-CoV-2 IgG antibody status, psychological factors, and age with menstrual irregularities among the larger sample (including vaccinated, unvaccinated, and unknown vaccination status participants). All independent variables met the criteria for linearity per the Box-Tidwell test. The model had a non-significant value on the Hosmer-Lemeshow test, which is suggestive of good model fit. There was no evidence of interactions (*X*^2^ = 8.59, *df* = 4, *p* = 0.07). There was no indication of multicollinearity issues in the final model, as the largest CNI = 16.34 and VPD = 0.32. Furthermore, age, perceived stress, depression, and loneliness did not confound the relationship of SARS-CoV-2 IgG antibodies to menstrual irregularities. After adjusting for age and psychological variables, the estimated odds of having irregular menstrual cycles were 7.03 times (95% CI [1.39, 35.60]; *p* = .019) higher among women with detectable IgG antibodies compared to women with undetectable IgG antibodies. Age was not associated with menstrual irregularities (aOR = 0.98; 95% CI [0.90, 1.07]; *p* = .670). In addition, there were no associations found between perceived stress (aOR 1.10; 95% CI [0.85, 1.43]; *p* = .465), depression (aOR 0.93; 95% CI [0.77, 1.12]; *p* = .425), or loneliness (aOR 1.11; 95% CI [0.59, 2.08]; *p* = .750) and menstrual irregularities.

## Discussion

This study is the first to use a biopsychosocial approach to examine the dual associations between biological and psychological factors and menstrual irregularities among reproductive age women during the COVID-19 pandemic. Overall, findings suggest there is a relationship between having a detectable history of SARS-CoV-2 IgG infection and/or COVID-19 vaccination and menstrual irregularities. Few women in our sample had been vaccinated for COVID-19 at the time they were tested for SARS-CoV-2 IgG, which suggests SARS-CoV-2 infection itself may be related to menstrual irregularities.

The current study did not find relationships between psychological variables and menstrual irregularities, which is supported by some prior studies and in conflict with others [20–22, 27]. It is possible discrepant findings are due to unique sociodemographic distributions across different study samples and different measures of menstrual irregularities. Women may also respond differently to questions about menstrual irregularities based on prior menstrual education, which was not evaluated in this study.

The current study supports growing research suggesting menstrual irregularities are associated with SARS-CoV-2 infection, potentially through biological mechanisms. It has been postulated that SARS-CoV-2 may infect endometrial tissue resulting in menstrual irregularities, but further research of the biological mechanisms is needed [28]. Women’s concerns about menstruation, fertility, and vaccination may drive vaccine hesitancy; as such, it is important to provide accurate information on the mechanisms underlying menstrual irregularities during the COVID-19 pandemic [41, 42].

Examining the link between SARS-CoV-2 infection and menstrual irregularities could deepen our understanding of immunology of the female reproductive system and how viral infections may affect reproductive health. Menstrual irregularities can signify altered functioning of the female reproductive system and obstruct women’s use of fertility awareness methods to prevent or promote conception [43]. Menstrual irregularities may also interfere with women’s ability to identify early stages of pregnancy, which could limit options for prenatal care or terminating pregnancy. Further research is needed to explore the clinical implications of the association of SARS-CoV-2 infection with menstrual irregularities and identify if infection impacts long-term reproductive health outcomes or characteristics of menstruation that lower quality of life, such as menstrual pain, menstrual distress, or rates of unintended pregnancies. It may be beneficial for women engaging in condomless intercourse to use contraceptives and receive regular pregnancy testing during and after SARS-CoV-2 infection. In addition, post-acute sequela of COVID may be more prevalent among women and manifested as menstrual irregularities [9, 44].

Prior to the COVID-19 pandemic, Black adults were more likely to experience post-traumatic stress disorder (PTSD) compared to white adults, and it is critical to continue investigating how social factors, such as discrimination or resource disparities, affect mental health and reproductive health among women from underserved racial and ethnic groups in the context of the pandemic [45]. As severe illness and unexpected deaths of loved ones during the COVID-19 pandemic may be a source of PTSD, future studies may seek to examine the role of bereavement and PTSD in influencing menstruation.

This study is limited in that the sample is small and may be underpowered to detect significant relationships. We were underpowered to examine differences in menstruation and other key factors across the 12 months of enrollment. It is possible that contextual factors, such heightened incidence of infection by SARS-CoV-2 variants, may impact findings. Additionally, a whole blood rapid antibody test was performed, which did not allow the differentiation between SARS-CoV-2 infection or COVID-19 vaccination, or assess the time to infection/vaccination as a potential factor that may influence menstruation as previously suggested [19]. Qualitative research may provide a more nuanced understanding of how women’s experiences influence mental health and menstruation.

We anticipate our study is generalizable to sexually active women of reproductive age in the United States whose menstrual cycles are not impacted by an intrauterine device, hormonal birth control, or a recent pregnancy. However, due to our small sample and the quickly changing nature of the pandemic, additional future research on menstrual outcomes is warranted and should include social constructs that may affect reproductive health such as race, ethnicity, or income [17].

Future research should employ more comprehensive measures of menstrual irregularities, including lifetime history and characteristics of menstrual irregularities, which we were unable to measure in the current study. Research is also needed to examine complex relationships between covariates linking menstrual irregularities and COVID-19. For example, menstrual irregularities have been associated with low nutritional intake and high energy expenditure, which can occur among athletes and individuals with disordered eating, regardless of body weight. Menstrual irregularities also correlate with obesity, although this may be due to the impact of metabolic and hormonal disorders on both menstruation and weight [13]. Dietary intake, adiposity, and body mass index (BMI) have been linked with COVID-19 disease incidence and severity [46–48]. As such, the relationship between COVID-19 and menstrual irregularities may be partially explained by nutritional status, physical activity, and body composition. Research to investigate these links must consider not only weight and BMI, but also eating behaviors, physical activity, nutritional intake, adiposity, and hormonal and metabolic disorders.

Overall, menstruation is an indicator of health often overlooked in infectious disease research. The association of SARS-CoV-2 infection with menstrual irregularities highlights the critical need for rigorous research evaluating menstruation during the COVID-19 pandemic to better understand how viral infection, stress, and mental health may impact women’s health.
